# Illuminating Blurry Vision: Visualization of Corneal Protein Deposition With Immunofluorescence in Two Illustrative Case Reports

**DOI:** 10.1155/crip/2915592

**Published:** 2026-02-20

**Authors:** Sena Zengin, Chaow Charoenkijkajorn, David Warner, T. David Bourne, Daisy Alapat, Neriman Gokden, Murat Gokden, Stephen Nix

**Affiliations:** ^1^ Department of Pathology, University of Arkansas for Medical Sciences, Little Rock, Arkansas, USA, uams.edu; ^2^ Department of Ophthalmology, University of Arkansas for Medical Sciences, Little Rock, Arkansas, USA, uams.edu; ^3^ Department of Pathology, Arkana Laboratories, Little Rock, Arkansas, USA; ^4^ Department of Medical Education, Alice L. Walton School of Medicine, Bentonville, Arkansas, USA

**Keywords:** crystalline keratopathy, immunofluorescence, LCD-1, MGUS

## Abstract

**Background:**

Monoclonal gammopathy of undetermined significance (MGUS) is an asymptomatic, premalignant disease with a progression rate of 0.5%–1% per year to multiple myeloma. It can rarely present with significant ocular symptoms in the context of crystalline keratopathy, necessitating medical and surgical interventions. Lattice corneal dystrophy Type I (LCD‐1), a rare inherited disorder caused by mutations of *TGFBI*, manifests with amyloid deposition within the corneal stroma and causes visual impairment. Here, we pictorially highlight protein deposition using immunofluorescence in two representative cases, both having undergone penetrating keratoplasty for blurry vision.

**Methods:**

Medical records were reviewed. Hematoxylin and eosin, special staining, immunohistochemistry, and immunofluorescent techniques were performed. A literature review was performed.

**Results:**

Case 1: Eosinophilic accumulations of the cornea were highlighted with PAS‐D and IgG‐kappa by immunohistochemistry, whereas immunofluorescence (IF) technique demonstrated IgG‐kappa (2+) staining in the stroma with rare globules in the epithelium. Case 2: Amorphous, eosinophilic deposits within the corneal stroma were congophilic with apple‐green birefringence on polarized light. Thioflavin T highlighted the amyloid through immunofluorescence. Mass spectrometry detected a peptide profile consistent with ATGFBI‐type amyloid deposition.

**Conclusion:**

Immunofluorescence can be helpful in the workup of corneal protein deposition, such as MGUS‐related crystalline keratopathy and LCD‐1.

## 1. Introduction

Monoclonal gammopathy of undetermined significance (MGUS) is a clinically silent plasma cell disorder that is often discovered by routine laboratory tests. It has a 0.5%–1% annual risk of progression to multiple myeloma (MM). Previous estimates using serum protein electrophoresis and immunofixation electrophoresis (SPEP/IFX) reported a ~3% prevalence in individuals aged over 50 years and more recently, mass spectrometry‐based screening of high‐risk individuals detected disease in close to 13% of patients aged 50 years or older [1, 2]. Although the disease does not require treatment, it can rarely present with significant ocular symptoms in the context of crystalline keratopathy, necessitating multiple medical and surgical treatments [1, 2].

Lattice corneal dystrophy Type I (LCDI), a rare autosomal‐dominant inherited disorder caused by mutations of *transforming growth factor beta-induced* (*TGFBI)*, manifests with amyloid deposition within the corneal stroma and can result in recurrent corneal erosions, corneal opacification, and visual impairment. Multiple treatments, including penetrating keratoplasty, may be needed [3].

Crystalline deposits have been investigated by electron microscopy, and immunohistochemistry is often helpful in clinical practice for characterizing the deposits. We show that immunofluorescence techniques can be helpful for characterizing deposits in the clinical setting. Specifically, we pictorially highlight protein deposition using immunofluorescence in a case of a 66‐year‐old man with MGUS and blurry vision (Case 1) and an 80‐year‐old woman with unexplained blurry vision (Case 2), both patients having undergone penetrating keratoplasty.

## 2. Methods

After institutional review board approval and determination as not human research (IRB 297569), the patients′ medical records were reviewed to gather relevant clinical data. Histologic analysis was conducted using hematoxylin and eosin, special staining, immunohistochemistry, and immunofluorescence. A comprehensive literature review was performed to contextualize and support the findings. No identifying information or images were included in this report. The findings in this paper were previously presented as an abstract at the American Association of Neuropathologists [4].

Formalin‐fixed paraffin‐embedded (FFPE) tissues were utilized for these analyses. For immunofluorescence, the following reagents were used: kappa light chains/FITC and lambda light chains/FITC, both polyclonal rabbit antihuman FITC‐conjugated antibodies, obtained from Agilent (Dako) with a titer of 1:10. Furthermore, antihuman IgA FITC, antihuman IgG FITC, and antihuman IgM FITC, all low F/P direct tag antibodies, were procured, each with a volume of 2 mL and sourced from goats. These reagents enabled the precise detection and visualization of specific proteins within the tissue samples.

## 3. Case Presentations

### 3.1. Case 1

A 66‐year‐old man with a past medical history of hepatitis C first presented to the ophthalmology clinic with several months of blurry vision and irritation in the right eye. He also reported a feeling of pressure behind the right eye and intermittent episodes of redness that resolved with antihistamine drops. Slit‐lamp examination revealed diffuse granularity and a crystalline appearance in both eyes.

An initial corneal biopsy was performed at the time but proved inconclusive. However, the clinical findings raised suspicion of a systemic disease such as MGUS, MM, and renal disease. Further laboratory work‐up revealed an M component of 1.4 g/dL, total protein of 8.2 g/dL, IgG of 2190 mg/dL (reference range [RR]: 700–1600), IgA of 138 mg/dL, and IgM of 188 mg/dL. The kappa‐free light chain was 3.8 mg/dL, the lambda‐free light chain was 1.8 mg/dL, and the kappa/lambda‐free light chain ratio was elevated at 2.1 (RR: 0.26–1.65). Bone marrow examination revealed 5% plasma cells without aberrant immunophenotype, leading to a diagnosis of IgG‐kappa MGUS.

During follow‐up 6 years later, the patient developed several complications: herpes simplex virus epithelial keratitis in the right eye, which left superficial scarring; bullous keratopathy with toxic keratitis in the left eye; periodic bilateral episcleritis/scleritis; and bilateral nuclear sclerosis. Despite treatment, the patient reported recurrent eye pain and progressive vision loss.

The patient underwent penetrating keratoplasty and a corneal transplant in the right eye, followed by the same procedure in the left eye in February 2023. The pathology report for the left corneal excision demonstrated PAS‐D‐positive and IgG kappa‐restricted eosinophilic globules in the cornea along with scarring, consistent with the patient′s history of MGUS. Immunofluorescent staining also revealed IgG‐kappa (2+) staining in the stroma, with rare globules in the epithelium, further supporting the diagnosis of crystalline keratopathy (Figure 1).

Figure 1MGUS‐associated crystalline keratopathy. Eosinophilic accumulations of the cornea were highlighted with PAS‐D (a) and IgG‐kappa by immunohistochemistry (b). IgG lambda immunohistochemistry and Congo red were negative (not illustrated). The immunofluorescence (IF) technique demonstrated IgG‐kappa (2+) staining in the stroma with rare globules in the epithelium (c). Lambda (1+) was largely limited to the stroma (d) as was IgG (1‐2+) and IgM (1+).

**Figure:**
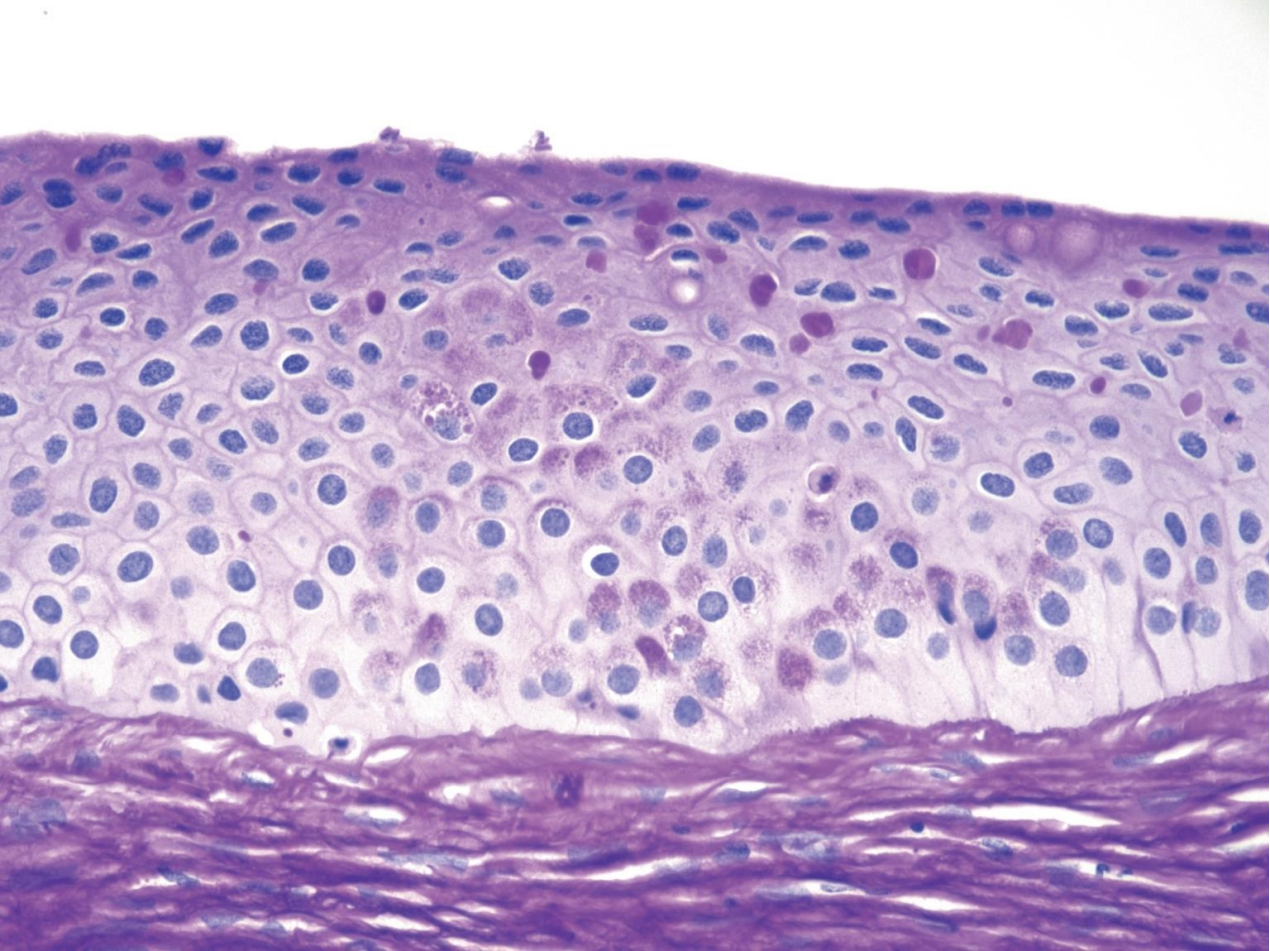
(a)

**Figure:**
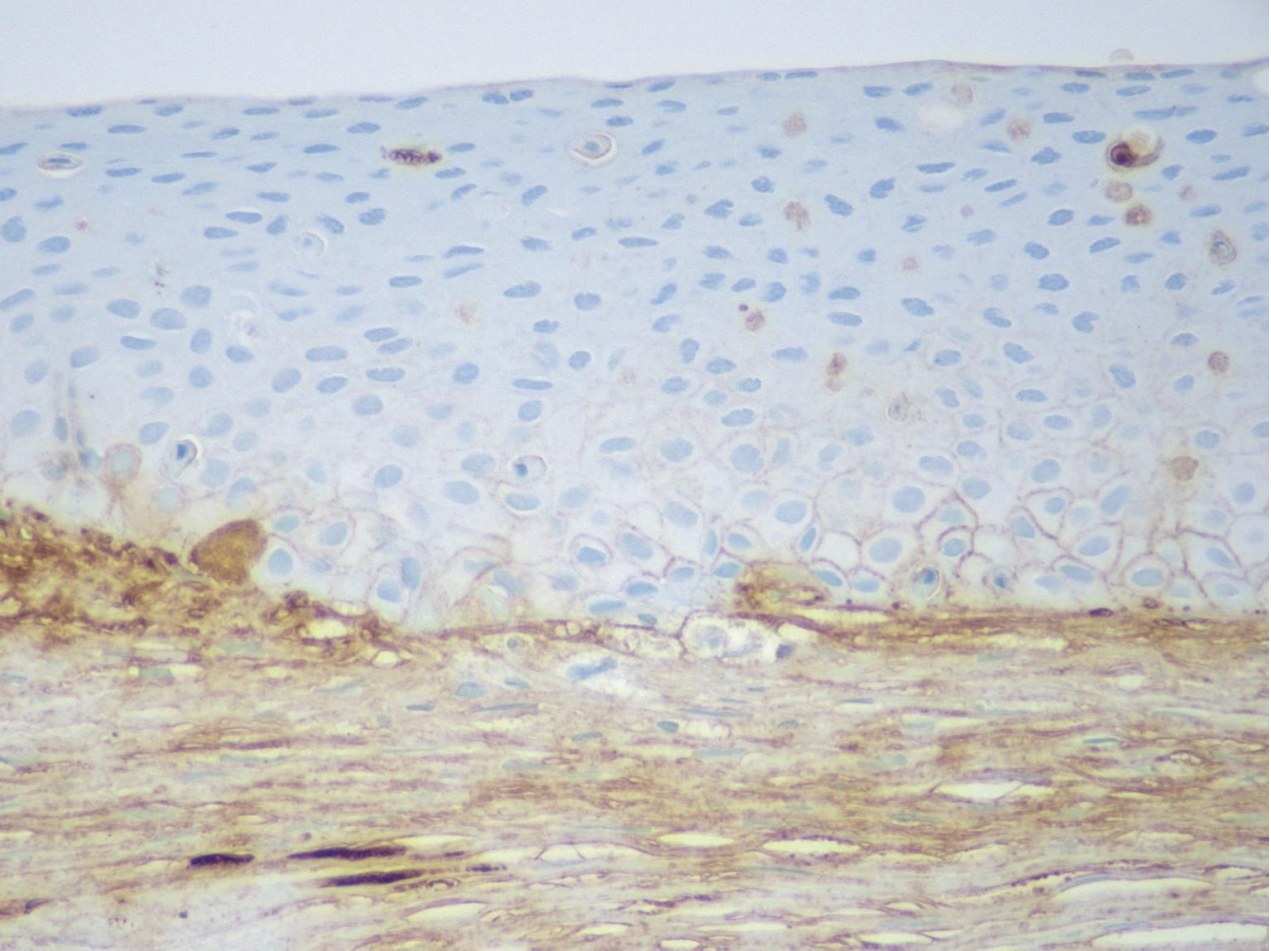
(b)

**Figure:**
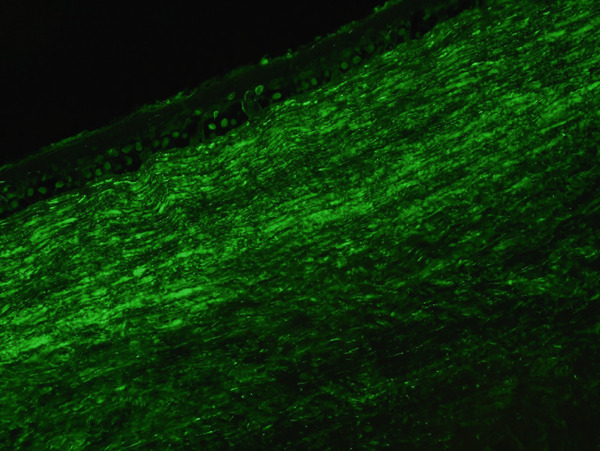
(c)

**Figure:**
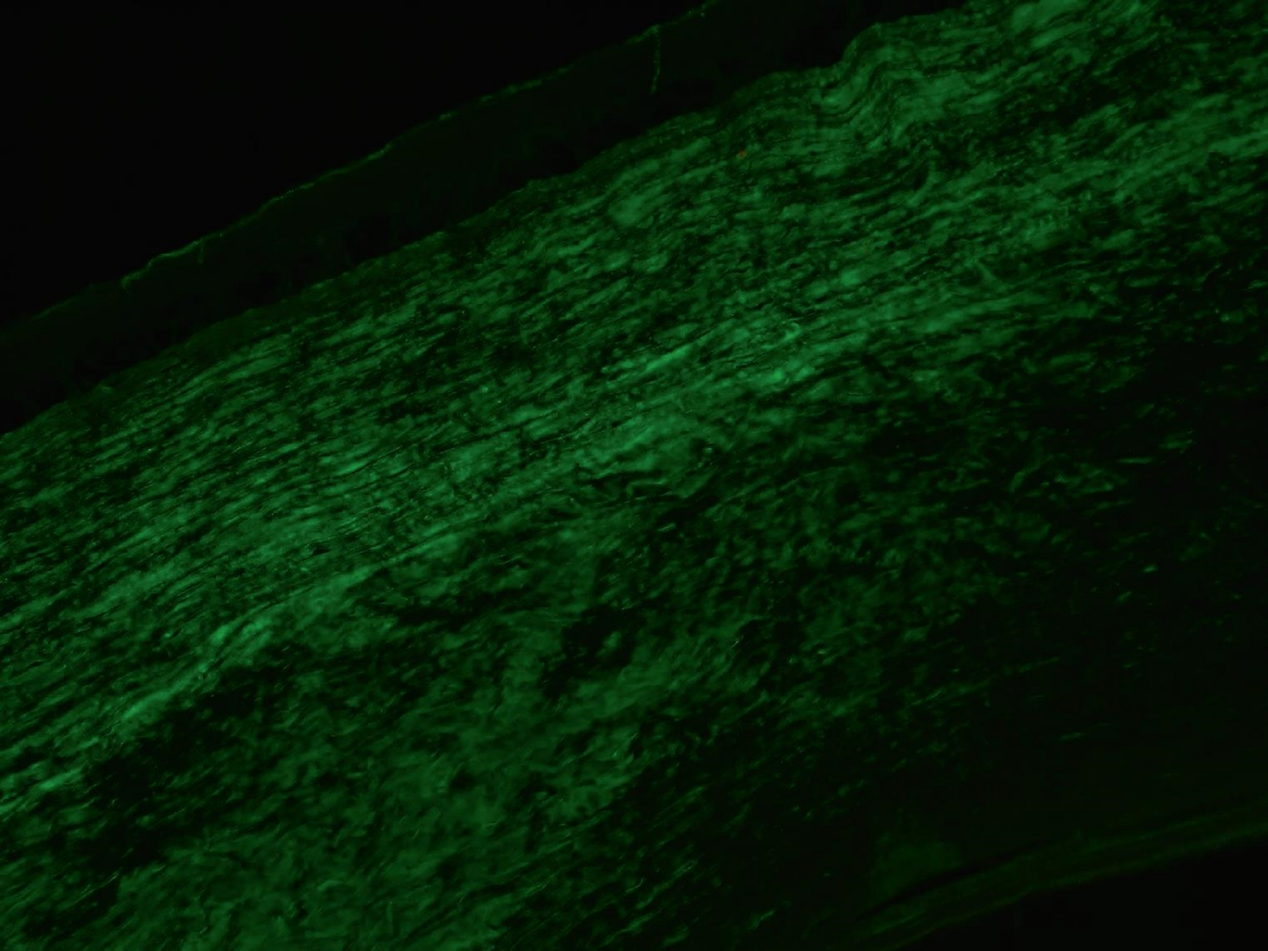
(d)

### 3.2. Case 2

An 80‐year‐old woman with hypertension and a past ocular history including high myopia and normal‐tension glaucoma, presented with slowly progressive vision loss in her left eye. The slit‐lamp examination showed irregular scattered vesicular lesions, involving Descemet membrane and endothelium, as well as dot‐like posterior stromal opacities with subtle diffuse ground‐glass opacification centrally.

Specular microscopy images showed corneal endothelial dropout (guttae) formations with scalloped edges (Figure 2). The diagnosis of posterior polymorphous corneal dystrophy (PPMD) was given, and because most of the pathology was located at the level of Descemet membrane, endothelial keratoplasty was offered.

Figure 2Specular microscopy images show corneal endothelial dropout (guttae) with scalloped edges in the left (a) and right eyes (b), most clearly seen in panel (b). Although this finding is typically associated with posterior polymorphous dystrophy (PPMD), the final pathologic diagnosis was determined to be lattice corneal dystrophy Type I (LCDI).

**Figure:**
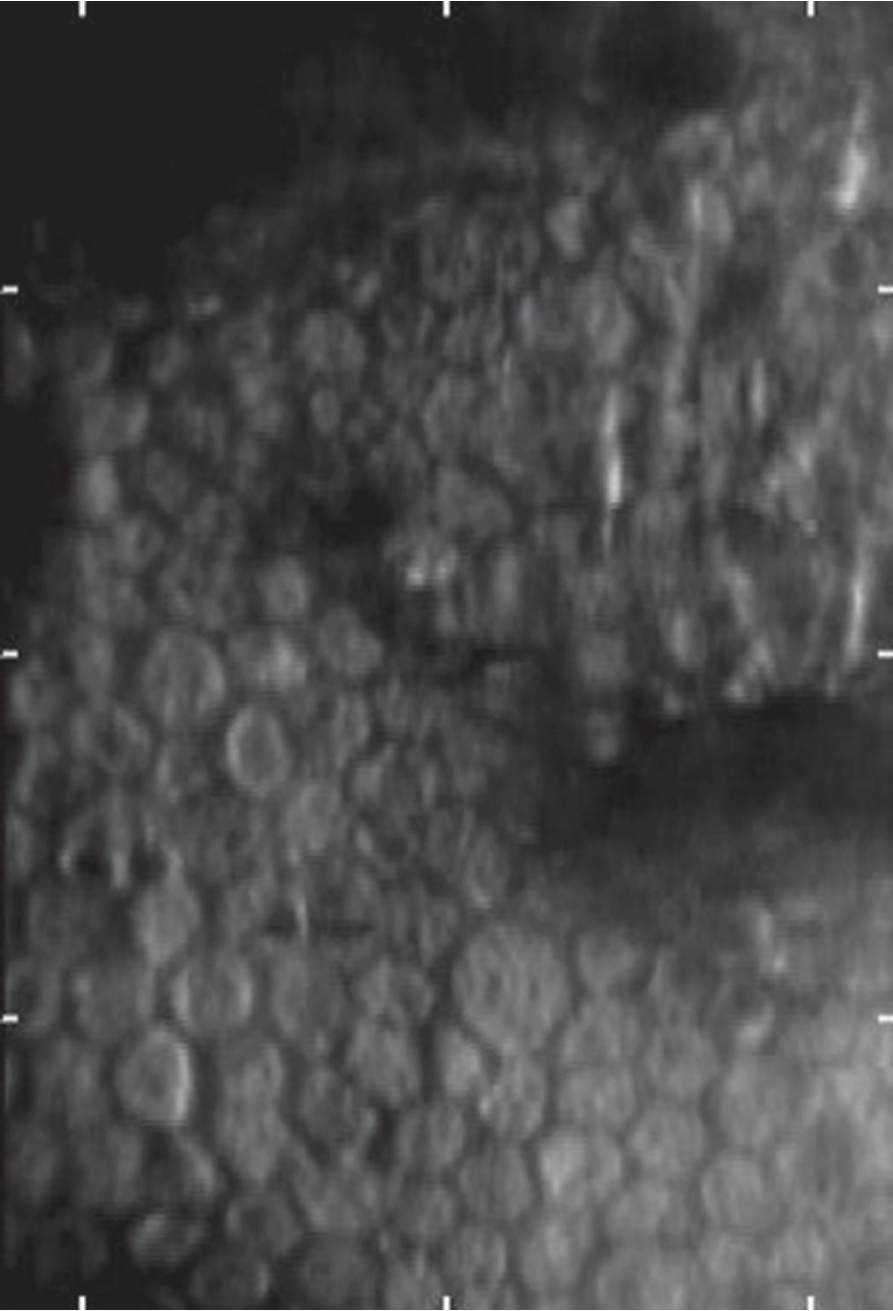
(a)

**Figure:**
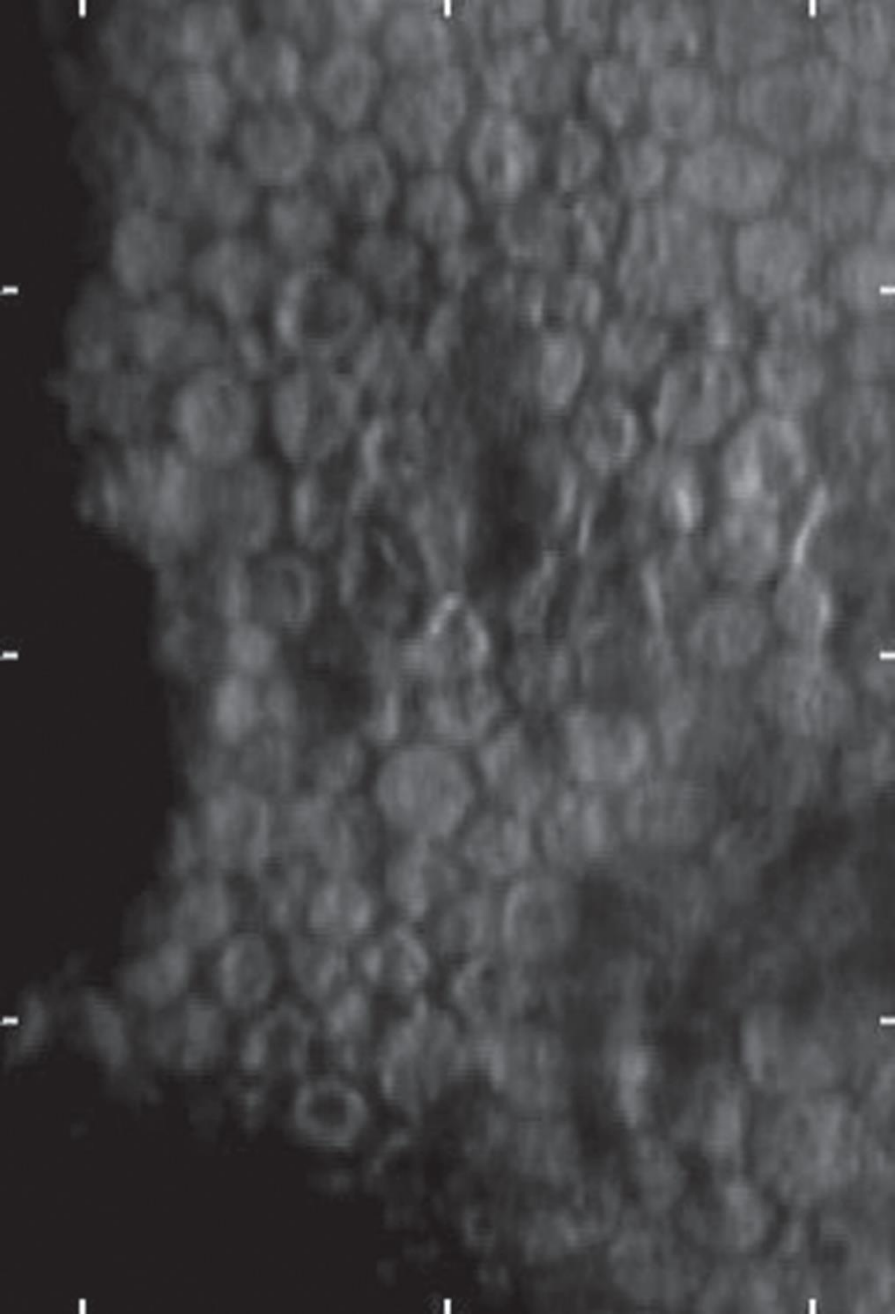
(b)

The patient underwent Descemet membrane endothelial keratoplasty but quickly developed graft failure and persistent corneal edema. Further consideration was then given to the potential visual significance of the stromal opacities, and penetrating keratoplasty was performed in lieu of repeating the endothelial keratoplasty on the left eye.

Histologic evaluation of the corneal tissue revealed amorphous, eosinophilic deposits within the stroma. Congo red staining demonstrated apple‐green birefringence in these deposits, consistent with amyloid, which was further highlighted by Thioflavin T (ThT) on immunofluorescence (Figure 3). Mass spectrometry identified a peptide profile consistent with TGFBI‐type amyloid deposition. This constellation of findings led to the diagnosis of LCDI.

Figure 3Lattice corneal dystrophy Type 1 corneal amyloid deposition. Amorphous, eosinophilic deposits within the corneal stroma (a) were congophilic (b) with apple‐green birefringence on polarized light (c). Thioflavin T highlighted the amyloid through immunofluorescence (d). Mass spectrometry detected a peptide profile consistent with ATGFBI‐type amyloid deposition (not illustrated).

**Figure:**
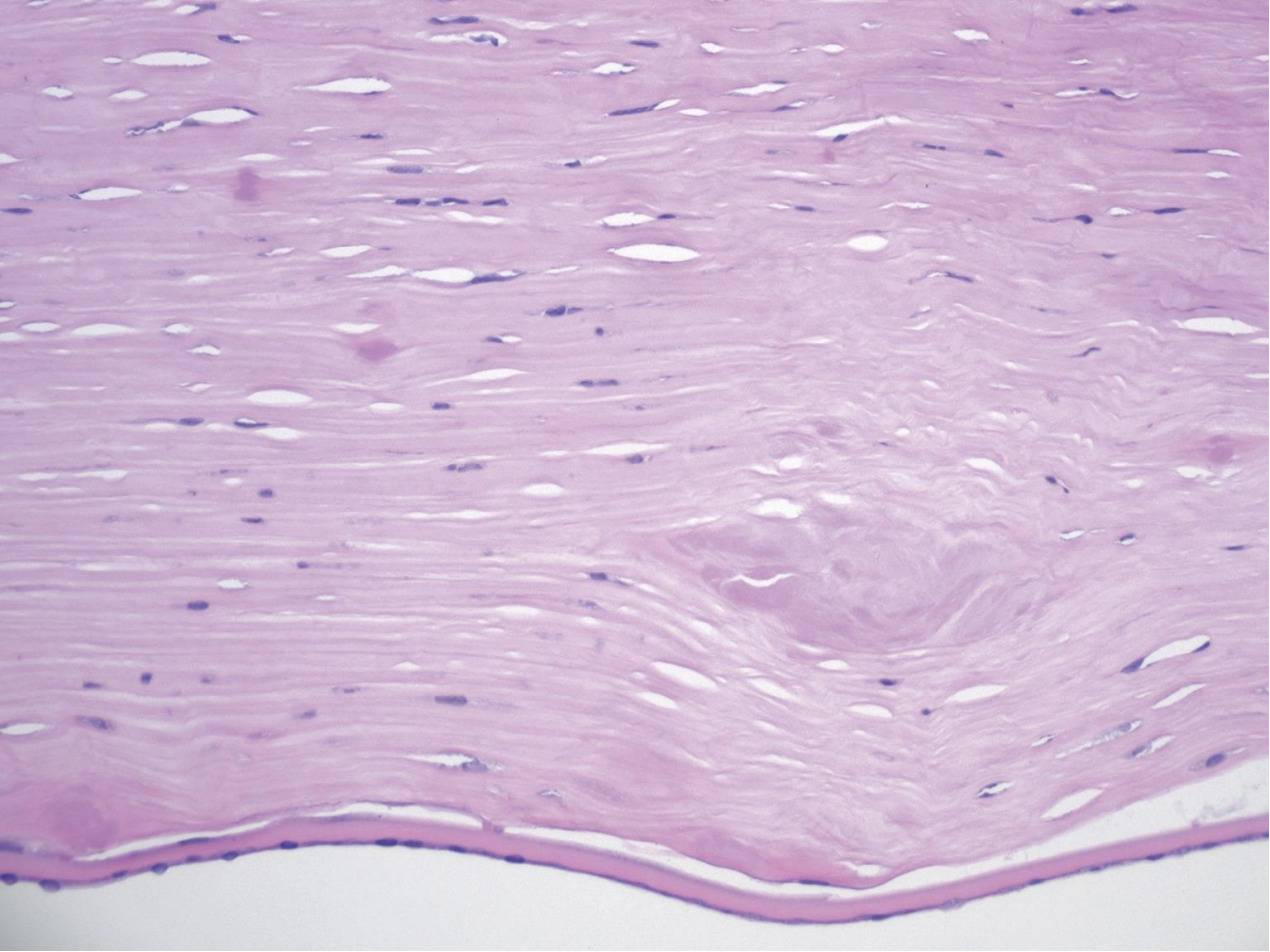
(a)

**Figure:**
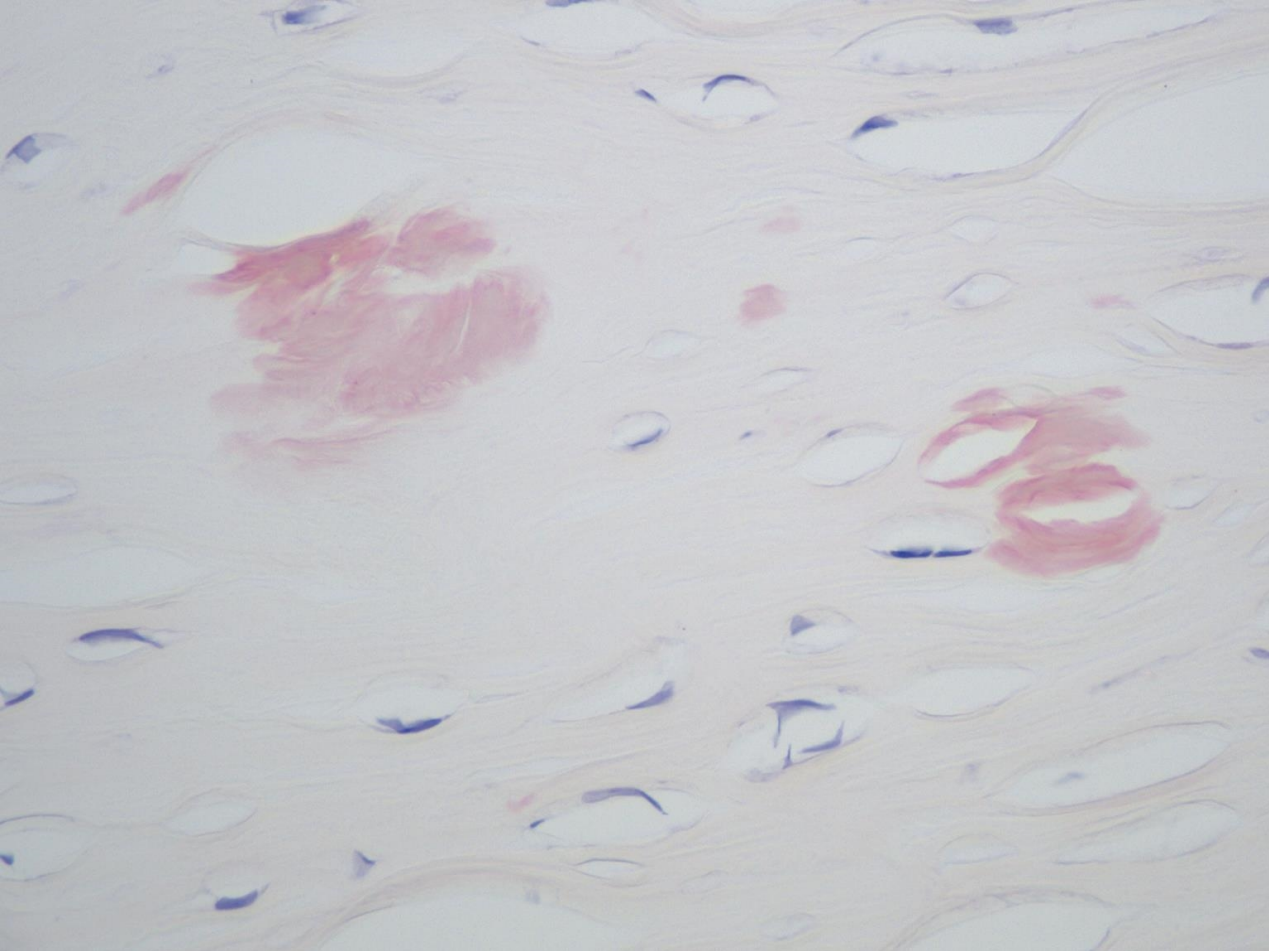
(b)

**Figure:**
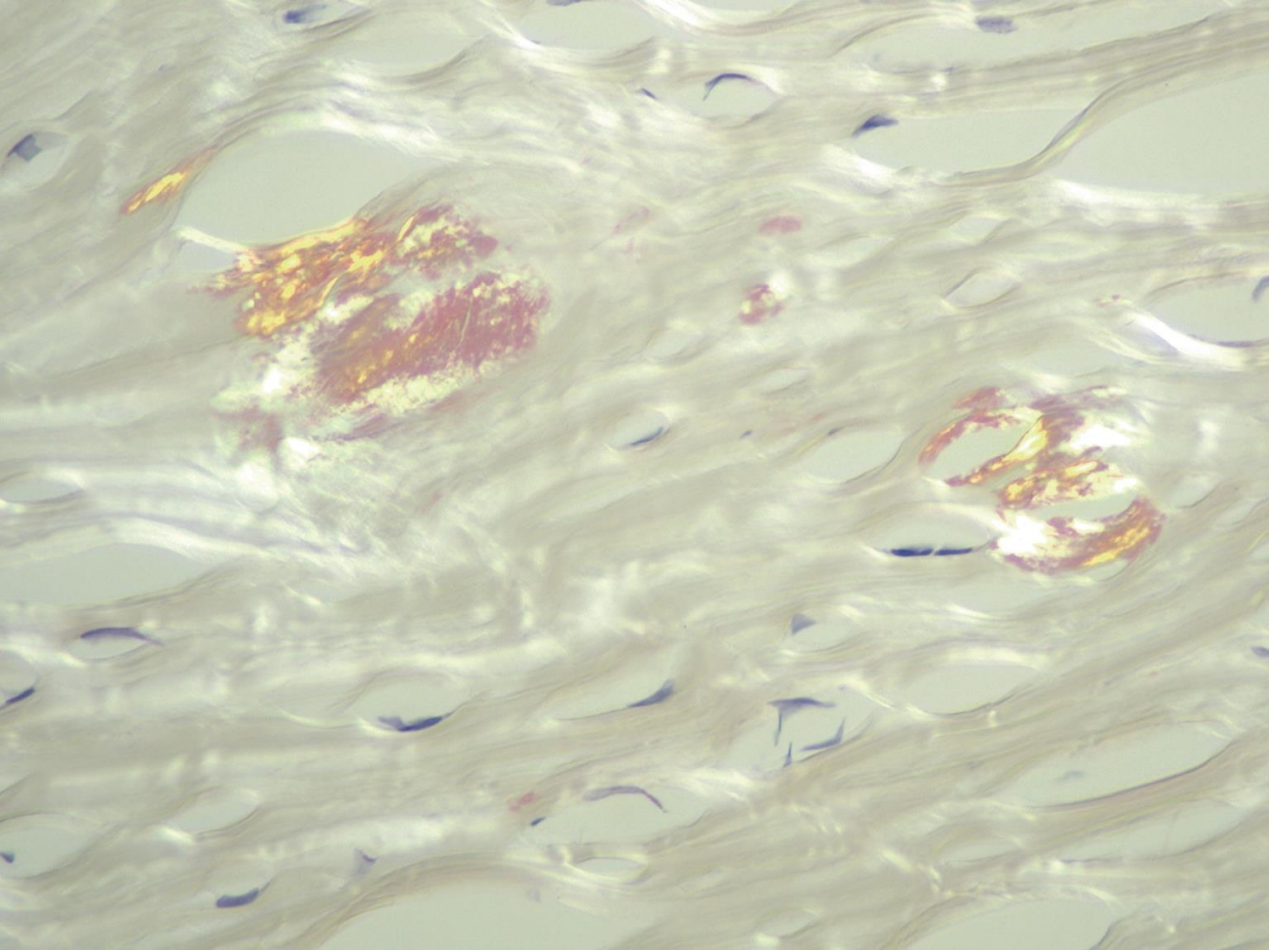
(c)

**Figure:**
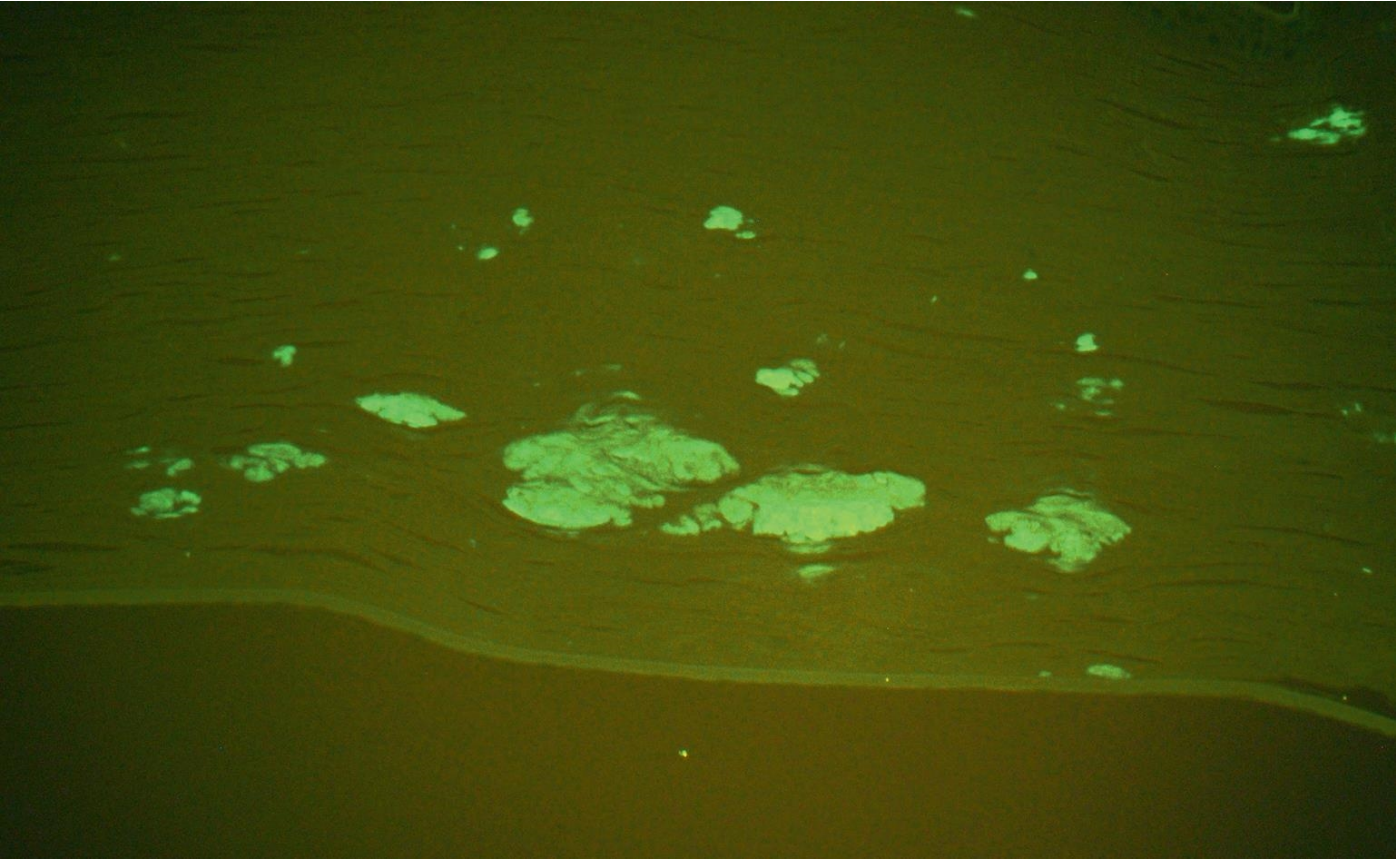
(d)

## 4. Discussion

### 4.1. MGUS‐Associated Paraproteinemic Keratopathy (PPK)

Corneal crystalline keratopathies encompass a broad spectrum of disorders characterized by the accumulation of various substances within the corneal layers, leading to blurriness, visual impairment, and ocular discomfort. Among these, MGUS‐related crystalline keratopathy is a notable, yet rare entity where abnormal monoclonal proteins deposit within the cornea, often presenting as fine, needle‐like crystals that can compromise corneal clarity and function [5, 6]. The prevalence of crystalline PPK has been rarely documented, with Bourne et al. reporting crystalline keratopathy in one of 100 patients with confirmed MM [3]. Despite the rarity of this entity, the differential diagnoses should include systemic paraproteinemias in patients presenting with corneal deposits and corneal opacification, as corneal manifestations can be the initial symptoms of systemic disease.

The morphological spectrum of PPK is notably diverse, prompting efforts to establish systematic classification systems. Lisch et al. first introduced a morphological framework in 2012, outlining five distinct patterns, which was later expanded in 2016 to include 17 subtypes based on corneal clouding morphology. The study proposed a refined framework for MGUS‐associated PPK, integrating clinicopathologic findings with previously published data [7].

The abnormal protein deposition occurs in various layers of the cornea, including the corneal epithelium, stroma, Bowman′s membrane, Descemet′s membrane, limbal vascular endothelium, conjunctiva, and lens. It may be either intracellular or extracellular, and depositions can appear in variable configurations (e.g., hexagonal, rectangular, or micro‐tubular) when examined under electron microscopy [7–10].

The mechanisms underlying corneal deposit formation in patients with MGUS remain unclear, and it is not well understood why certain immunoglobulin molecules undergo crystallization within tissues. Crystalloids have been proposed to originate from multiple potential sources, including tears, limbal blood vessels, aqueous humor, and stromal keratocytes. Using immunoelectron microscopy, Henderson et al. identified four types of IgG‐kappa crystalloids within the corneal stroma, epithelium, and limbal vascular endothelium, and the investigators proposed that these deposits arise primarily from circulating immunoglobulins that traverse damaged limbal microvasculature [11]. Another study reported increased corneal light scattering in the central cornea along with hyperreflective keratocytes by using in vivo confocal microscopy, and these findings may indicate monoclonal gammopathy [10].

### 4.2. Corneal Dystrophies

Corneal dystrophies represent a heterogeneous group of inherited disorders that typically manifest bilaterally and symmetrically, are slowly progressive and are not related to systemic factors. These dystrophies are classified based on the affected corneal layer, and the nature of the deposits as well as divided into epithelial and subepithelial dystrophies (e.g., epithelial basement membrane dystrophy), epithelial‐stromal TGFBI dystrophies (e.g., lattice corneal dystrophy ([LCD]), stromal dystrophies (e.g., macular corneal dystrophy), and endothelial dystrophies (e.g., PPMD) according to International Classification of Corneal Dystrophies (IC3D) [12].

LCDI is characterized by the deposition of amyloid fibrils. The amyloid deposition is usually found in the anterior and mid‐stroma, but interestingly, in our patient it was located more posteriorly. The deposition distorts the architecture of corneal lamellae and is characteristically positive with Congo red and shows apple‐green birefringence under polarized light. LCDI is associated with mutations in the *TGFBI* gene, leading to the formation of dots and lattice‐like opacities in the corneal stroma. These lines usually start centrally and superficially, spreading centrifugally and deeply though presentations can vary [13, 14].

Symptoms in LCDI include ocular discomfort, pain, and visual impairment due to frequent recurrent erosive attacks, often resulting in a marked decrease in vision [12]. Although LCDI usually affects both eyes, unilateral involvement has also been described [15, 16]. Interestingly, in our case [2], the patient was thought to have PPMD, which usually presents as an asymmetric disease and tends to involve the Descemet membrane and endothelium but was proven otherwise after histologic examination as described above.

Corneal dystrophies are generally diagnosed through clinical examination, detailed patient history, and advanced imaging techniques such as slit lamp biomicroscopy and corneal topography. Genetic testing and molecular analysis also play a crucial role in identifying specific types of corneal dystrophies.

Although immunofluorescence techniques have typically not been employed in diagnosing corneal dystrophies, several studies reported immunofluorescence findings on selected corneal dystrophies have been reported, including LCD, Reis–Bücklers′ corneal dystrophy, and macular corneal dystrophy [17–19]. Another study on granular and lattice deposits corneal dystrophy caused by R124C mutation of *TGFBI*, likely categorized as a variant of formerly known Avellino corneal dystrophy (ACD), which is currently classified as granular corneal dystrophy Type 2 (GCD2), demonstrated the potential application of ThT with immunofluorescence to detect amyloid deposits of TGFBI. The authors also suggested that granular and lattice deposits can be associated with R124C *TGFBI* mutation, which is usually associated with LCD [17] as opposed to the classically associated phenotype of R124H in ACD (GCD2) [20]. They argue against the idea of TGFBI‐related corneal dystrophies solely classified based upon specific gene mutations; rather, single gene mutations can hold multiple phenotypes [17].

### 4.3. Evaluation of Corneal Protein Deposition and Immunofluorescence

Electron microscopy findings have been widely described for protein deposition in crystalline keratopathies. However, given the limited availability of electron microscopy in many centers, immunohistochemistry and immunofluorescence are helpful for a prompt diagnosis. Although direct immunofluorescence on frozen tissue has been the norm for the evaluation of immune complexes [21–24], there are disadvantages related to processing, such as the need for thick sections of fresh frozen tissue and diffuse antigen distribution [25]. Nasr et al. showed that immunofluorescence techniques on formalin‐fixed, paraffin‐embedded tissue yield comparable results to those obtained on frozen sections for most pathogenic immunoglobulins and complements [26]. Similarly, our cases emphasize and pictorially illustrate the findings of the immunofluorescence technique by using FFPE tissue. We highlight that immunofluorescence on FFPE tissue can practically contribute to the diagnosis of monoclonal crystalline keratopathies and corneal dystrophy, specifically for LCDI and MGUS‐related PPK.

The application of immunofluorescence on FFPE tissue has advantages and disadvantages. Advantages include the ability to perform immunofluorescence analysis when frozen tissue samples are unavailable [27], superior preservation of tissue morphology compared with corresponding frozen tissue, potential unmasking of target antigens not detected by routine immunofluorescence on frozen tissue samples [28], and the ability to apply the technique to archival FFPE material without the need for long‐term frozen storage. Disadvantages include potentially decreased detection sensitivity due to degradation of target antigens in FFPE tissue [29] and a lack of some commercially available antibodies to use in FFPE tissue (e.g., antibodies to IgG subclasses).

## 5. Conclusion

In the realm of corneal protein depositions, a wide variety of diseases pose significant diagnostic challenges and can sometimes lead to detrimental outcomes requiring multiple treatments and surgeries. Protein deposition may indicate local pathologies, such as dystrophies or systemic disease, necessitating further investigation. Immunofluorescence analysis can highlight immunoglobulins and amyloid proteins, and this technique can be a practical tool for investigating corneal protein deposition, providing a helpful workup strategy for practicing pathologists as shown in these pictorial examples of IgG‐kappa corneal deposition in a case of MGUS crystalline keratopathy and amyloid corneal deposition in LCDI.

## Funding

No funding was received for this manuscript.

## Ethics Statement

This study was determined as not human research by institutional review board (IRB 297569), and no identifying information or figures is included in this report.

## Conflicts of Interest

The authors declare no conflicts of interest.

## Supporting information


**Supporting Information** Additional supporting information can be found online in the Supporting Information section. Formalin‐fixed paraffin‐embedded tissues are used: kappa light chains/FITC, concentrate—polyclonal rabbit antihuman, FITC‐conjugated antibody, immunofluorescence, Ig fraction, 2 mL, Vendor—Agilent (Dako), and Titer—1:10; lambda light chains/FITC, concentrate—polyclonal rabbit antihuman, FITC‐conjugated antibody, immunofluorescence, Ig fraction, 2 mL, Vendor—Agilent (Dako), and Titer—1:10; antihuman IgA FITC, low F/P‐4—direct tag (Kent Laboratories, 2 mL, goat); antihuman IgG FITC, low F/P‐4.6—direct tag (Kent Laboratories, 2 mL, goat); antihuman IgM FITC, low F/P‐4.7—direct tag (Kent Laboratories, 2 mL, goat).

## Data Availability

The data that support the findings of this study are available from the corresponding author upon reasonable request.
