# Supplementary material for: Illuminating Blurry Vision: Visualization of Corneal Protein Deposition With Immunofluorescence in Two Illustrative Case Reports
Source: Case Rep Pathol. 2026 Feb 20;2026:2915592. doi: 10.1155/crip/2915592 (PMC12921536; doi:10.1155/crip/2915592)
Supplement: Supplementary file 1 — Supporting Information Additional supporting information can be found online in the Supporting Information section. Formalin‐fixed paraffin‐embedded tissues are used: kappa light chains/FITC, concentrate—polyclonal rabbit antihuman, FITC‐conjugated antibody, immunofluorescence, Ig fraction, 2 mL, Vendor—Agilent (Dako), and Titer—1:10; lambda light chains/FITC, concentrate—polyclonal rabbit antihuman, FITC‐conjugated antibody, immunofluorescence, Ig fraction, 2 mL, Vendor—Agilent (Dako), and Titer—1:10; antihuman IgA FITC, low F/P‐4—direct tag (Kent Laboratories, 2 mL, goat); antihuman IgG FITC, low F/P‐4.6—direct tag (Kent Laboratories, 2 mL, goat); antihuman IgM FITC, low F/P‐4.7—direct tag (Kent Laboratories, 2 mL, goat). [file CRIP-2026-2915592-s001.docx]

**Supplemental Information**

Formalin-fixed paraffin-embedded tissues are used.

**Kappa** Light Chains/FITC, Concentrate. Polyclonal Rabbit Anti-Human, FITC-conjugated antibody, Immunofluorescence, Ig fraction, 2 mL, Vendor - Agilent (Dako), Titer - 1:10.

**Lambda** Light Chains/FITC, Concentrate. Polyclonal Rabbit Anti-Human, FITC-conjugated antibody, Immunofluorescence, Ig fraction, 2 mL, Vendor- Agilent (Dako), Titer - 1:10.

Anti Human IgA FITC, Low F/P - 4 - Direct Tag (Kent Laboratories, 2mL, goat)

Anti Human IgG FITC, Low F/P-4.6- Direct Tag (Kent Laboratories, 2mL, goat)

Anti Human IgM FITC, Low F/P-4.7- Direct Tag (Kent Laboratories, 2mL, goat)
